# An android can show the facial expressions of complex emotions

**DOI:** 10.1038/s41598-024-84224-3

**Published:** 2025-01-19

**Authors:** Alexander Diel, Wataru Sato, Chun-Ting Hsu, Alexander Bäuerle, Martin Teufel, Takashi Minato

**Affiliations:** 1 Clinic for Psychosomatic Medicine and Psychotherapy, LVR-University Hospital Essen, University of Duisburg-Essen, 45147 Essen, Germany; 2 Guardian Robot Project, RIKEN, Kyoto, Japan; 3 Center for Translational Neuro- and Behavioral Sciences (C-TNBS), University of Duisburg-Essen, 45147 Essen, Germany

**Keywords:** Affective computing, Android, Dynamic face emotion expressions, Secondary emotions, Human behaviour, Mechanical engineering

## Abstract

Trust and rapport are essential abilities for human–robot interaction. Producing emotional expressions in the robots’ faces is an effective way for that purpose. Androids can show human-like facial expressions of basic emotions. However, whether androids can show the facial expression of complex emotions remains unknown. In this experiment, we investigated the android Nikola’s ability to produce 22 dynamic facial expressions of complex emotions. For each video, 240 international participants (120 Japanese, 120 German) rated the emotions expressed by Nikola. For 13 complex emotions (i.e., amusement, appal, awe, boredom, contentment, coyness, hatred, hesitation, moral disgust, not face, pain, sleepiness, suspicion), participants of both samples rated the target emotion above the mean of other non-target emotions. Four emotions (bitterness, confusion, pride, relief) were rated above mean by one sample. For twelve of these emotions, target emotions were among the highest ranked. The results suggest that androids can produce the facial expressions of a wide range of complex emotions, which can facilitate human–robot interactions.

## Introduction

Social robots, defined as robots designed for social interaction with humans, find increasing use in society, for example as companions for elders^1,2^, hospital patients^3^, individuals on the autism spectrum^4^, and children^5^. Social robots can also take on service roles like hospitality or waitering^6^, and replace human actors use in situations threatening human health, such as during the COVID-19 pandemic^7^. Thus, social robots find various societal uses that focus on human interaction.

Robots with the ability to express emotions are perceived as active social interaction partners^8^. A robots can express emotions through tone of voice, phrases, gestures, symbols, or facial expressions, depending on its technical capabilities. Especially facial expressions are a powerful tool to communicate a wide range of information on one’s own internal states^9^.

A recently developed android called Nikola has been shown to be able to show the humanlike facial expressions of six basic emotions (i.e., anger, disgust, fear, happiness, sadness, and surprise)^10^. Nikola has 35 pneumatic actuators in the head to imitate humanlike facial actions. The researchers^10^ evaluated Nikola’s actions using the Face Action Units System (FACS)^11^, a system developed to define facial expression based on the activation of face action units (AUs) which are groups of facial muscles used to define facial expressions, and validated the 17 human AUs. In a psychological experiment, the researchers showed the photographs of Nikola’s facial expressions of six basic emotions, and participants recognized all emotions higher than chance level. Further, the clips of Nikola’s dynamic facial expressions of basic emotions were appropriately recognized as in humans’ expressions. These results validate that the android Nikola can produce humanlike facial expressions of basic emotions.

However, whether androids can show facial expressions beyond basic emotions remains unknown. Several previous psychological studies have revealed that humans’ facial expressions of emotions can show not only basic or primary emotions but also complex or secondary emotions, depicting their specific AU patterns^12,13^. Although there is debate on the adequate categorization of emotions, many emotions beyond the basic ones play a crucial role in social interaction (e.g., pride, shame, amusement, awe, spite) and have been associated with patterns of facial expressions^14–20^. A previous study revealed that virtual human agents can simulate a wide set of facial emotion expressions^21^. Based on these data, together with the android Nikola’s ability to reliably imitate human AUs^10^, we hypothesized that the android can show the facial expressions of complex emotions.

We tested this hypothesis in a psychological experiment testing human participants’ ratings of Nikola’s facial expression clips. By referring to previous literature defining human facial expression patterns using the FACS^14–20^, we programmed Nikola to express 22 facial expressions beyond basic emotion expressions, including amusement, appal, awe, bitterness, boredom, confusion, contentment, coyness, desire for food, embarrassment, flirt, hatred, hesitation, moral disgust, not face, pain, pride, relief, scorn, shame, sleepiness, and suspicion. Complex emotions were chosen based on the results of a literature search on the currently published literature focusing on complex emotions defined via AUs. Complex emotions were selected if at least one published study validated the respective AUs for the expression (see Table 3). We investigated whether participants can correctly attribute the expressions above chance. For each of the 22 facial expressions, we tested the following prediction:

Nikola’s facial expression is rated higher to express the target emotion compared to non-target emotions.

The hypotheses are tested on samples of two populations (participants recruited in Japan and Germany) to investigate cross-cultural validity of Nikola’s emotion expressions.

## Results

### Japanese sample

**Fig. 1:**
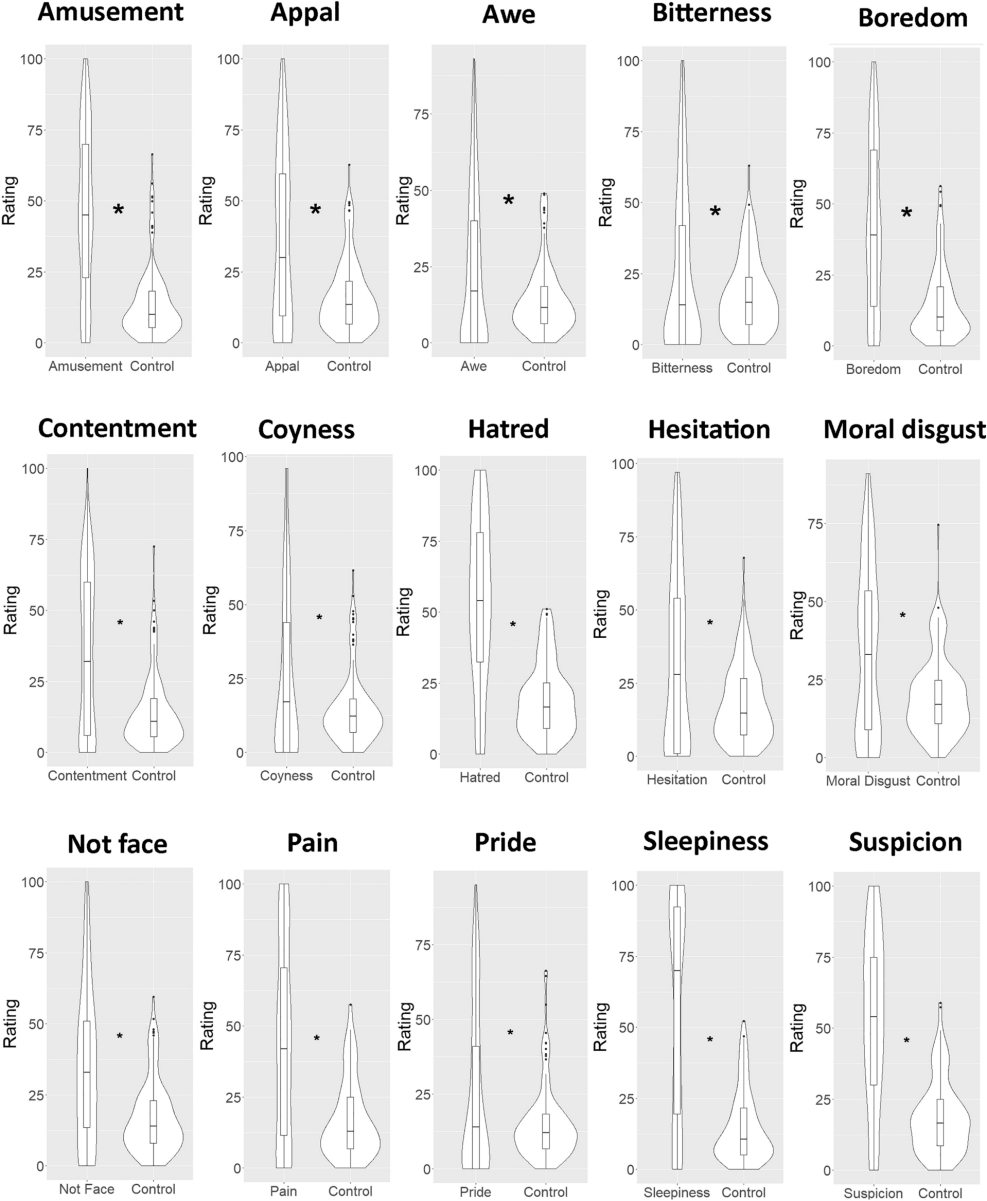
Significant differences between ratings of the target emotion and the other non-target emotions for the Japanese sample. Violin plots show rating distributions while boxplots show median and Q1 to Q3 range of the data Error bars indicate 95% confidence intervals. Asterisks indicate significant difference between the target vs non-target emotions.

The results of *t*-tests are summarized in Table 1*.* Significant results are depicted in Fig. 1.

**Table 1:** Test statistics of one-tailed comparisons between the emotion expression rating of the target emotion and the mean of all other non-target emotions in a Japanese sample.

Emotion expression	*t*-value	*p*-value	Cohen’s *d*
Amusement	*t*(119) = 12.83	< .001*	1.44
Appal	*t*(119) = 8.61	< .001*	0.88
Awe	*t*(119) = 3.49	< .001*	0.46
Bitterness	*t*(119) = 4.11	< .001*	0.37
Boredom	*t*(119) = 9.60	< .001*	1.16
Confusion	*t*(119) = 0.55	.291	0.03
Contentment	*t*(119) = 8.13	< .001*	0.95
Coyness	*t*(119) = 4.28	< .001*	0.51
Desire for food	*t*(119) = 1.314	.096	0.10
Embarrassment	*t*(119) = -11.66	> .999	1.09
Flirt	*t*(119) = -7.80	> .999	0.91
Hatred	*t*(119) = 14.80	< .001*	1.53
Hesitation	*t*(119) = 6.34	< .001*	0.64
Moral disgust	*t*(119) = 6.53	< .001*	0.68
Not face	*t*(119) = 6.69	< .001*	0.86
Pain	*t*(119) = 8.18	< .001*	1.03
Pride	*t*(119) = 4.16	.001*	0.44
Relief	*t*(119) = 3.24	.007	0.32
Scorn	*t*(119) = 1.06	.146	0.14
Shame	*t*(119) = -18.84	> .999	1.79
Sleepiness	*t*(119) = 10.80	< .001*	1.47
Suspicion	*t*(119) = 13.55	< .001*	1.53

*Note*. Asterisks indicate significant difference between the target vs non-target emotions. Bonferroni-adjusted α-level of 0.0023 (i.e., 0.05/22 comparisons) was used to evaluate significant *p*-values.

In summary, for the Japanese sample, the ratings of 15 target emotions (i.e., amusement, appal, awe, bitterness, boredom, contentment, coyness, hatred, hesitation, moral disgust, not face, pain, pride, sleepiness, suspicion) out of 22 were significantly higher than the mean of other non-target emotions (*t* > 2.92, Bonferroni-adjusted *p* < 0.05), with effect sizes ranging from medium (*d* = 0.37 for bitterness) to high (*d* = 1.53 for suspicion and hatred). The ratings of seven expressions (i.e., confusion, desire for food, embarrassment, flirtiness, relief, scorn, shame) did not significantly differ from the mean of other non-target emotions.

### German sample

**Fig. 2:**
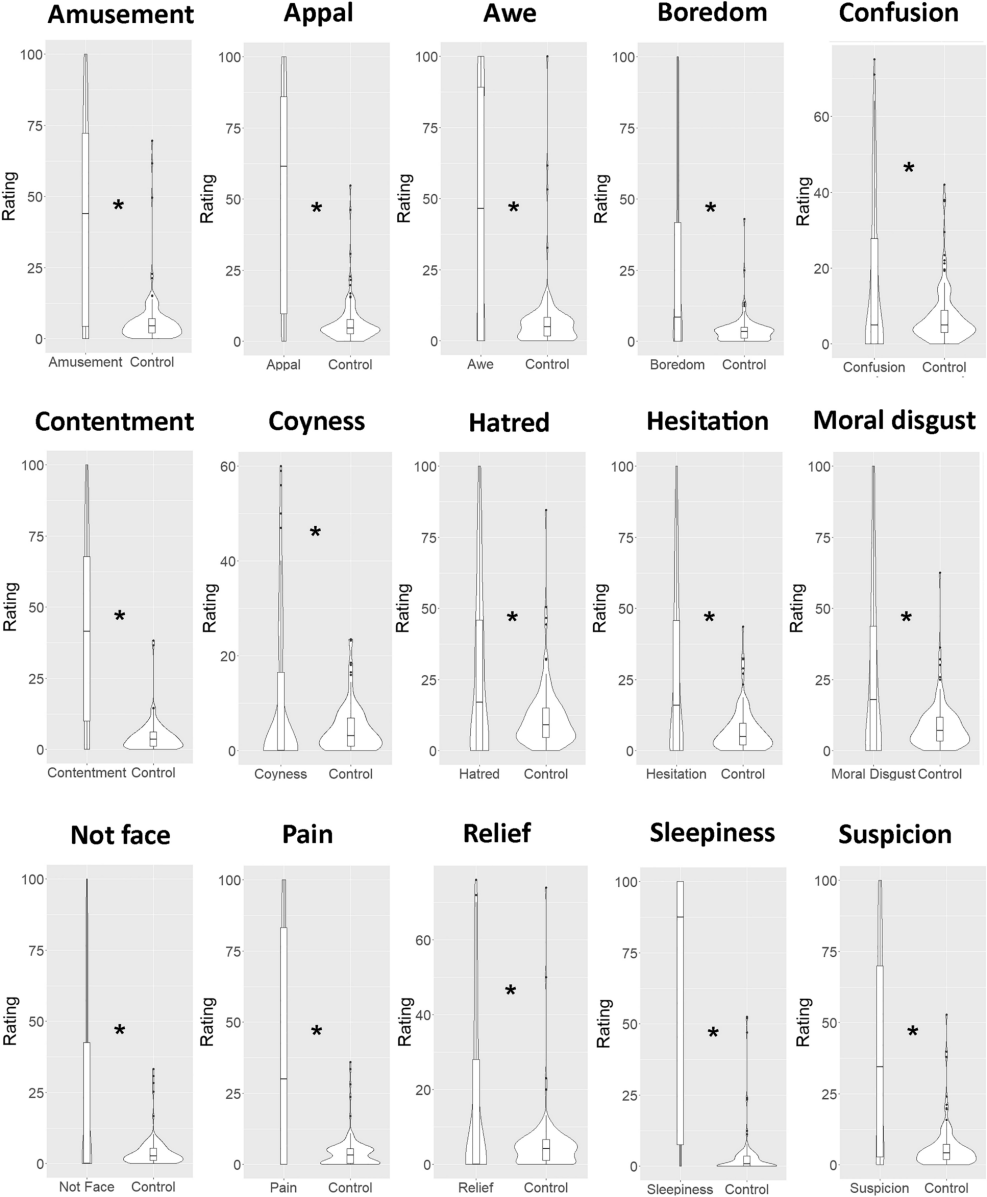
Significant differences between ratings of the target emotion and the other non-target emotions for the German sample. Violin plots show rating distributions while boxplots show median and Q1 to Q3 range of the data. Error bars indicate 95% confidence intervals. Asterisks indicate significant difference between the target vs non-target emotions.

The results of *t*-tests are summarized in Table 2. Significant results are depicted in Fig. 2*.*

**Table 2:** Test statistics of one-tailed comparisons between the emotion expression rating of the target emotion and the mean of all other non-target emotions in a German sample.

Emotion expression	*t*-value	*p*-value	Cohen’s *d*
Amusement	*t*(119) = 12.36	< .0001*	1.45
Appal	*t*(119) = 12.88	< .0001*	1.62
Awe	*t*(119) = 10.57	< .0001*	1.38
Bitterness	*t*(119) = 0.480	.316	− 0.02
Boredom	*t*(119) = 7.33	< .0001*	0.91
Confusion	*t*(119) = 4.86	< .0001*	0.57
Contentment	*t*(119) = 13.07	< .0001*	1.58
Coyness	*t*(119) = 3.30	.0006*	0.44
Desire for food	*t*(119) = -17.17	> .999	− 1.31
Embarrassment	*t*(119) = -9.92	> .999	− 1.08
Hatred	*t*(119) = 6.55	< .0001*	0.71
Hesitation	*t*(119) = 7.73	< .0001*	0.91
Moral disgust	*t*(119) = 7.20	< .0001*	0.79
Not face	*t*(119) = 6.31	< .0001*	0.82
Pain	*t*(119) = 9.74	< .0001*	1.30
Pride	*t*(119) = -2.83	.997	− 0.31
Relief	*t*(119) = 5.78	< .001*	0.64
Scorn	*t*(119) = 0.50	.310	0.03
Shame	*t*(119) = -12.59	> .999	− 1.62
Sleepiness	*t*(119) = 15.74	< .001*	1.98
Suspicion	*t*(119 ) = 10.36	< .001*	1.29

*Note*. Asterisks indicate significant difference between the target vs non-target emotions. Bonferroni-adjusted α-level of 0.0023 (i.e., 0.05/22 comparisons) was used to evaluate significant *p*-values.

In summary, the German sample could rated 15 complex emotions (amusement, appal, awe, boredom, confusion, contentment, coyness, hatred, hesitation, moral disgust, not face, pain, relief sleepiness, suspicion; *t* > 2.92, Bonferroni-adjusted *p* < 0.05) as the target emotions compared to the average non-target emotion ratings, with effect sizes ranging from medium (*d* = 0.44 for coyness) to high (*d* = 1.98 for sleepiness).

A target emotion being rated significantly above the average of non-target emotions indicates a general tendency towards correctly identifying the expression. However, if a target emotion is still not rated among the highest ratings, and especially if the highest rated emotion is a conceptually distinct emotion, then this would question the validity of the expression. Therefore, a second post-hoc criterion on correct attribution of emotion is introduced: the target emotion should be among the highest ranked emotions for an expression, as indicated as being either (1) the top-ranked emotion, or (2) not differing significantly from the top-ranked emotion. To further evaluate accurate emotion attribution, post-hoc analyses have been conducted to evaluate whether target emotions had the highest ratings (or were among the highest rated) emotions for each expression. Results are summarized in Table 3.

**Table 3:** Summary of the ranking of each target emotion and significance test compared to the ratings of the highest-ranked non-target emotion (if the target emotion was not highest ranked), divided by expression and sample.

Expression	Japanese		German	
	Rank	Highest ranked emotion (significance)	Rank	Highest ranked emotion (significance)
Amusement	1*		2*	Contentment (*t*(110) = 0.48, *p* = .803)
Appal	1*		1*	
Awe	4*	Amusement (*t*(119) = 5.69, *p* < .0001*)	1*	
Bitterness	7*	Scorn (*t*(119) = 4.14, *p* < .0001*)	6	Scorn (*t*(110) = 9.39, *p* < .0001*)
Boredom	2*	Sleepiness (*t*(119) = 1.07, *p* = .288)	2*	Sleepiness (*t*(119) = 4.19, *p* < .0001*)
Confusion	8	Hatred (*t*(116) = 7.8, *p* < .0001*)	4*	Suspicion (*t*(113) = 3.52, *p* = .0006*)
Contentment	2*	Amusement (*t*(119) = 3.51, *p* = .0006*)	1*	
Coyness	5*	Scorn (*t*(119) = 2.12, *p* = .036)	3*	Contentment (*t*(109) = 5.13, *p* < .0001*)
Desire for food	7	Amusement (*t*(117) = 12.06, *p* < .0001*)	8	Amusement (*t*(104) = 16.02, *p* < .0001*)
Embarassment	14	Scorn (*t*(103) = 9.81, *p* < .0001*)	7	Amusement (*t*(93) = 9.29, *p* < .0001*)
Flirtiness	16	Suspicion (*t*(105) = 7.24, *p* < .0001*)		
Hatred	1		3*	Scorn (*t*(119) = 4.62, *p* < .0001*)
Hesitation	1		2*	Embarassment (t(119) = 1.94, p = .054)
Moral disgust	6*	Hatred (*t*(119) = 6.78, *p* < .0001*)	3*	Scorn (*t*(119) = 5.64, *p* < .0001*)
Not face	2*	Suspicion (*t*(119) = 8.86, *p* < .0001*)	1*	
Pain	1*		2*	Sleepiness (*t*(119) = 0.4, *p* = .691)
Pride	4	Contentment (*t*(119) = 6.54, *p* < .0001*)	7	Suspicion (*t*(102) = 7.34, *p* < .0001*)
Relief	5*	Amusement (*t*(119) = 6.51, *p* < .0001*)	3*	Amusement (*t*(116) = 7.29, *p* < .0001*)
Scorn	10	Confusion (*t*(118) = 4.39, *p* < .0001*)	6	Appall (*t*(101) = 5.26, p < .0001*)
Shame	13	Bitterness (*t*(103) = 15.06, *p* < .0001*)	19	Suspicion (t(92) = 10.29, p < .0001*)
Sleepiness	1*		1*	
Suspicion	1*		1*	

Asterisks indicate whether the target emotion was significantly rated higher compared to the average (see Tables 1 and 2). If the target emotion was not ranked 1, the highest ranked non-target emotion is named and post-hoc significance tests were conducted on the difference in ratings between the target and top-ranked emotion.

Based on the extended results, emotion expressions are finally categorized according to the evidence supporting their validity in Table 4.

**Table 4:** Categorization of emption expressions’ validity according to the analyses. Emotions in brackets signify top-ranked non-target emotions if the target emotions was not highest ranked.

Categorization	Target emotion (and highest ranked non-target emotion)
Top-ranked rated target emotion in both samples	Appal, sleepiness, suspicion
Top-ranked rated target emotion in one sample; non-significant difference from top-ranked non-target emotion in the other sample	Amusement (contentment), hesitation (embarrassment), pain (sleepiness)
Top-ranked rated target emotion in one sample; significant difference from top-ranked non-target emotion in the other sample	Awe (amusement), contentment (amusement), hatred (scorn), not face (suspicion)
Non-significant difference from top-ranked non-target emotion in other sample; significant difference from top-ranked non-target emotion in the other sample	Boredom (sleepiness), coyness (scorn/contentment)
significant difference from top-ranked non-target emotion in both samples	Bitterness (scorn), confusion (hatred/suspicion), moral disgust (hatred/scorn), relief (amusement)
Not significant above average	Desire for food (amusement), embarrassment (scorn/amusement), flirtiness (suspicion), pride (contentment/suspicion), scorn (confusion/appal), shame (bitterness/suspicion)

In summary, twelve expressions were among the highest ranked emotions in at least one sample, among them seven top-ranked in one sample (awe, contentment, hatred, not face, amusement, hesitation, pain) and three top-ranked in both samples (appal, sleepiness, suspicion). For emotions that were rated above averaged but differed significantly from the target emotion, most had a valence similar to the target emotion (bitterness and scorn, moral disgust and hatred/scorn, and relief and amusement), except for confusion whose valence differs from hatred/suspicion.

## Discussion

In summary, the results showed that the android Nikola could produce the facial expressions of complex emotions. Seventeen emotion expressions were rated as the correct expression by at least one sample (German or Japanese) while thirteen emotion expressions were correctly identified by both samples, relative to the ratings of the incorrect expressions. Post-hoc analyses further suggest that twelve out of these seventeen emotions were among the highest rated emotions in at least one sample. Taken together with the research on facial expressions of six basic emotions^10^, Nikola is thus capable of producing a variety of recognizable expressions. An android with the ability to reproduce recognizable human facial expressions can have a broad range of applicability in social robotics with a focus on emotional interaction, such as customer service, care, or nursing. Since Nikola’s expressions are not perceived as uncanny when compared to a human’s expressions^22,23^, Nikola is already bridging a limitation common for humanlike robots, namely the uncanny valley effect which may impair trust and affective interaction^24,25^.

Four of the expressions were rated higher as the target emotion compared to the non-target emotions by only one sample: Specifically, bitterness and pride for the Japanese sample, and confusion and relief for the German sample. The expression of emotions is culturally filtered, for example in their intensity^26–28^, which may lead to differences in the expression and thus ratings of emotion expressions. Furthermore, the effect sizes of these four expressions tended to be relatively low compared to the other expressions, indicating that those may be more difficult to recognize in general.

Even when target emotions significantly differed from the highest-ranked emotions, they nevertheless were conceptually similar: For example, bitterness was rated as scorn, moral disgust as hatred or scorn, and relief as amusement. These results suggest that even when Nikola’s expression is not optimally recognized, the valence of these expressions is nevertheless communicated. As emotion recognition can depend on other factors such as the social situation in which it is expressed^29^, the identification of the correct valence together with the appropriate social scenario may facilitate correct emotion recognition in a social setting.

Five out of Nikola’s facial expressions were not detected relative to the non-target emotions by either sample. For these expressions, the highest-rated non-target emotions also tended to be conceptually different from the target emotions (e.g., embarrassment was rated as amusement, flirtiness as suspicion, pride as contentment, scorn as confusion, or shame as bitterness), indicating further that the emotions were not attributed correctly. There may be several reasons as to why. First, Nikola may suffer from some hardware limitations for specific facial gestures. For example, the flirty face includes a kissing mouth which uses a high intensity of AU 18 (lip pucker) that Nikola’s hardware may be incapable of reproducing. In addition, the expression of scorn consists of looking down on the interaction partner while raising its head, which may be limited by Nikola’s ability to move its eyes down to a high degree. Furthermore, certain movements may have been interpreted incorrectly or may have obscured Nikola’s face. Second, the correct identification of emotion expressions also relies on social or multimodal context^29^ which was not present in this study. For example, the expression of embarrassment utilizes an appeasement smile^30^ which may have not been recognized as such (and instead as a regular smile) without social context. In addition, multimodal information (e.g., saying “ouch” for a pain face) may further enhance the ability to correctly identify the expression. Third, selected AUs may have been inadequate for the emotion expression: For the expression of confusion, only one study was found^19^, which may have provided insufficient information on the AUs necessary for confusion. However, such explanations are largely speculative and would need further research to investigate.

Our results also have implications for future research, such as Nikola being a valuable tool in face-processing research. Research on face perception is often limited by a lack of stimulus variety and the inability to easily control certain stimulus variables (e.g., speed or sequence of AU motion). Nikola can provide an embodied agent whose facial expression variables like AU sequence can be easily controlled, allowing the creation of facial expression stimuli that would be difficult to recreate with human expressions (e.g., for face AU motion asynchrony and configural processing^31^). Thus, Nikola allows an easy investigation of new research questions.

One limitation of the study is the controlled depiction of the emotion expressions. The videos lacked social and multimodal context which may further enhance the ability to identify the emotion expression. Further research may aim to provide social context (i.e., simulating a social setting to which the respective emotional reaction would be appropriate) or additional multimodal information (e.g., “ouch” sound when expressing pain).

Another limitation is the use of rating scales to assess emotion detection. In this study, emotion labels with relative proportions were used to assess detection. This method does not limit users’ response options to one, which has been criticized in other methods such as choice from array tasks^14^ which may be especially limiting with an increasing number of expressions or labels. However, this method still limits participants’ options to the labels presented. Although an emotional expression may be rated as such relative to the non-target emotions by a participant, this would not mean that the participant would rate the emotion correctly without verbal references. Future study may attempt to measure recognized emotions using other measures, such as choice from array or free labelling (albeit with a smaller selection of emotion expressions).

Furthermore, comparing the target emotion ranking to the average ratings would be distorted by the high dissimilarity of the expression with non-target emotions. Significant differences may then occur because of the dissimilarity of the non-target emotions rather than the similarity of the target emotion. Hence, the post-hoc analyses on the emotion rankings provide further information on if a target emotion was significantly different from the average because it was among the highest ranked emotions.

The correct identification of emotion expressions was here measured by verbal labels which are commonly used for face emotion rating tasks^10,14,16^. Such results may be further supported by indirect measures such as physiological or behavioral responses to facial emotion expressions. However, research on the physiological and behavioral responses to complex emotion expressions are too sparse to use specific validated responses for complex expressions. Future research may try to use Nikola’s expressions alongside a human’s expressions to investigate whether the android’s expression elicit similar responses.

## Conclusion

Social humanoid robots gain increasing importance and use in human life. To ensure trust-based interaction between humans and robots, display of affect is crucial. In this study, the android kid Nikola managed to produce 17 out of 22 facial expressions of complex emotions that can be rated high relative to the non-target expressions across an international sample. For twelve of these expressions, target emotions were among the highest ranked on at least one sample. Taken together with previous research^10^, Nikola is capable of simulating a wide range of human emotional facial expressions.

## Methods

### Participants

A total of 120 Japanese participants (59 women and 61 men; *M*_age_ = 34.12, *SD*_age_ = 5.58) and 120 German participants (67 women, 51 men, and two other; *M*_age_ = 35,13, *SD*_age_ = 11.74) were tested. An a priori power analysis using G*Power software ver. 3.1.9.2^32^ (Faul et al., 2007) with an α-level of 0.0032 (Bonferroni- adjusted level of 0.05 for 22 comparisons) and an effect size of *d* = 0.34 according to previous research on Nikola’s facial expressions (Sato et al., 2022), 120 participants were necessary to reach a power of 1-β = 0.8. The participants were recruited online through CrowdWorks (Tokyo, Japan) and Prolific (www.prolific.com), and needed a mean of 31.1 min to complete the study. The experiment was approved by the Ethics Committee of RIKEN and the Ethics Committee of University Duisburg-Essen (24-11870-BO), and performed in accordance with the Declaration of Helsinki.

### Material

#### Emotion expressions

Emotion expressions were selected through a literature research on secondary or complex emotion expressions whose relevant face AUs have been validated. Several research studies have been used to select the relevant AUs for each expression^14–20^. An overview of each emotion expression, a description provided for the participant during the study, and selected AUs is found in Table 5.

**Table 5:** An overview of the selected facial expressions (alphabetical order), their description, relevant AUs (and additional movements) used to program the expression, and reference literature.

Emotion	Description	Action units, misc. movements	Reference literature
Amusement	The feeling of being entertained or made to laugh	2, 6, 14	^14,18^
Appal	Strong feelings of shock, dismay, and disapproval	4, 10	^17^
Awe	Feeling of respect sometimes mixed with fear and surprise; an emotion combining dread, veneration, and wonder inspired by authority or by the sacred or sublime	1, 5, 12, 26	^17^
Bitterness	Feeling of anger and unhappiness; resentful anger and disappointment	6, 9, 24	^18^
Boredom	State of being bored; weary and restless through lack of interest	1, 43	^14^
Confusion	Lack of understanding; state of being perplexed, disoriented, or disconcerted	4, 5, 6, 7, 10, 12, 23	^19^
Contentment	State of being satisfied with one’s possessions, status, or situation; happiness or satisfaction because you have everything you need	12, 43, “deep breasth”	^14,16^
Coyness	Being shy or keeping something secret	6, 7, 12, 25, 54	^14,16^
Desire for food	Positive appraisal of delicious-looking food	7, 12, 25, “jaw dropped”	^14,16^
Embarrassment	Feeling of being ashamed or shy; a state of self-conscious confusion and distress	6, 7, 12, 25, 54, “control smile”	^14,16^
Flirty face	Playfully showing casual sexual interest or liking	6, 7, 12, 18, 25 (one-sided blink)	
Hatred	Extreme dislike or disgust	4, 10,	^17,18^
Hesitation	Pausing because of being uncertain or nervous; to hold back in doubt or indecision	1, 6, 7, 13, 14, 17, 20, eyes turn left	
Negative moral judgment	Communicating violation of one’s rights, beliefs, or norms	4, 7, 9, 10, 14, 17, 24	^15^
Pain	Mental or physical sensation of distress or suffering; feeling of suffering caused by injury or illness	4, 6, 7, 9, 10, 43	^14^
Pride	Pleasure, confidence, or satisfaction because of having done something good	7, 12, 53	^14,16^
Relief	Feeling of happiness after the removal of something oppressive, painful, or distressing	12, 18, 25, 43, “jaw drop”, “head down”	^16^
Scorn	Strong feeling of disrespect, dislike, and mockery	4, 11, 15, head up, eyes down	^18^
Shame	Uncomfortable feeling caused by consciousness of one’s own shortcoming or impropriety	4, 7, 54	^14,16^
Sleepiness	Feeling tired, wanting to sleep	2, 15, 17, 45, “jaw drop” (yawn)	^20^
Suspicion	Doubt or lack of trust	4, 7, 14 (unilateral)	^18^
“Not-face”/“thinking face”	Negation or disagreement	4, 7	^15^

*Note*. The expression of *flirtiness* was not used for the German sample due to ethical considerations of avoiding to associate an android depicting a child with sexually suggestive behavior.

#### Android

The android Nikola was used given his ability to imitate human face AUs and basic emotions. Nikola was developed at RIKEN for the purpose of studying human–robot emotional interactions^10^. Only the head part was used in this study. The robot head is human-like in appearance, similar to that of a male human child. It is approximately 28.5 cm in length and weighs approximately 4.6 kg. It has 35 actuators: 29 for facial muscle actions, 3 for head movement (roll, pitch, and yaw rotations), and 3 for eyeball control (panning of the individual eyeballs and tilting of both eyeballs). The movements are driven by pneumatic (air) actuators, and the entire surface, except for the back of the head, is covered in soft silicone skin. An extended system comprising control valves, an air compressor, and computers controls the actuators and sensors. In-house programs created using Python (Python Software Foundation, Fredericksburg, VA, USA) and ROS (Open Robotics, Mountain View, CA, USA) were used to control Nikola’s actuators. A previous study^10^ validated Nikola’s 17 AUs, including 1 (inner brow raiser), 2 (outer brow raiser), 4 (brow lowerer), 5 (upper lid raiser), 6 (cheek raiser), 7 (lid tightener), 10 (upper lip raiser), 12 (lip corner puller), 14 (dimpler), 15 (lip corner depressor), 16 (lower lip depressor), 18 (lip pucker), 20 (lip stretcher), 22 (lip funneler), 25 (lips part), 26 (jaw drop), and 43 (eyes closed). Nikola can show the unilateral activation of these AUs, as well as any head movements (e.g., AU 54: head down) and eyeball movements. Using the AU information in Table 1, Nikola was programmed to express each facial expression.

#### Stimuli

**Fig. 3:**
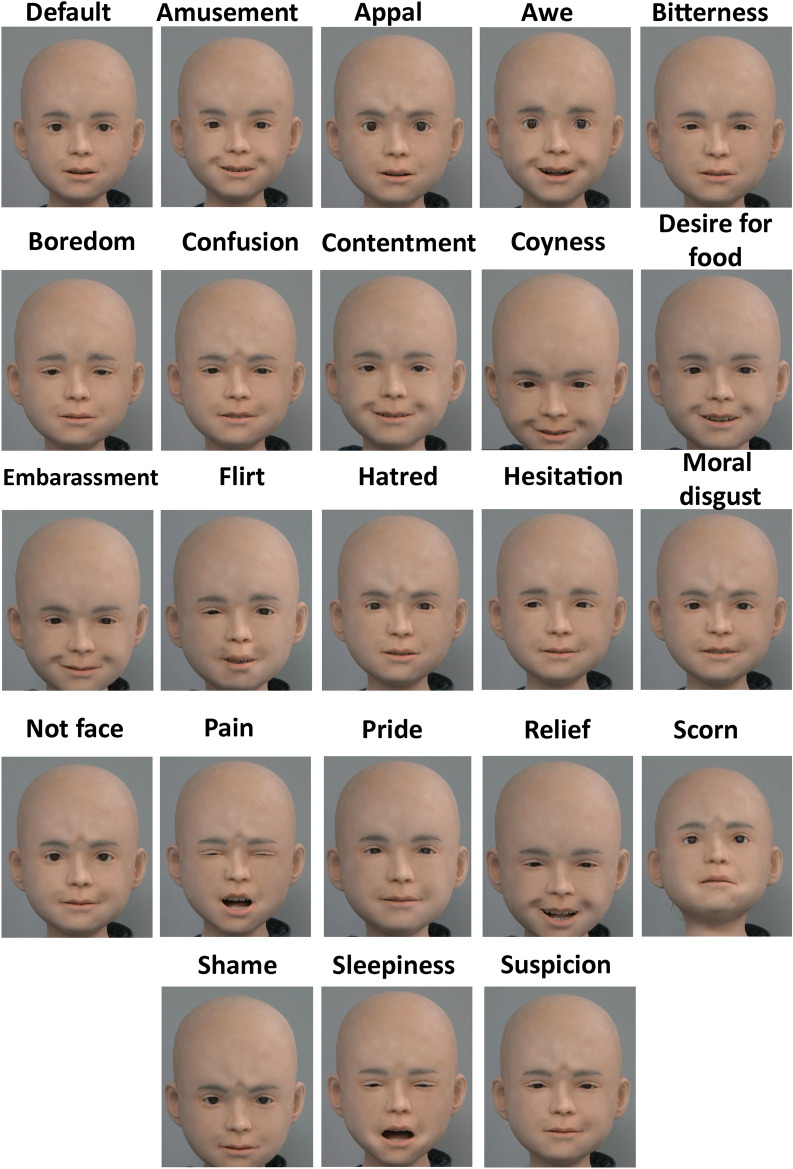
Nikola’s peak facial expressions including the default face followed by each expression in alphabetical order.

Frontal videos of Nikola were made for each expression. The videos were recorded using a digital web camera (HD1080P; Logitech, Tokyo, Japan). Each video lasted one second, began with a default face and ended at the peak (highest AU intensity) of the expression. Screenshot of each expression’s peak and the default face are seen in Fig. 3.

### Procedure

Participant received a link to the online survey. After reading the study information and providing informed consent, participants were shown the videos in a randomized sequential order. For each video, participants were given 22 scales ranging from 0 to 100, each named after one of the expressions and a description of the expression (see Table 1). Participants were tasked to rate the video based on to which degree each named emotion is expressed. All scales were shown simultaneously and each video could be repeated indefinitely.

### Data analysis

Data was analysed via RStudio (R ver. 4.1.2). Outlier removal was conducted at 1.5 times interquartile range at the participant level for each expression scale rating and facial expression. Out of 116,160 values (120 times 22 times 22 times 2), 10,092 outlier values were removed. One-tailed *t*-tests with Bonferroni-adjusted alpha values of 0.0023 (i.e., 0.05/22 comparisons) were conducted. For each test, the rating of the target emotion was compared to the mean rating of all other non-target emotions for a given stimulus. For the averaged non-target emotion, each participants’ ratings of the non-target emotions were averaged. Each t-test was paired (each participant’s target emotion rating compared to the participant’s averaged non-target emotion ratings) in order to allow within-subject comparisons. In addition, t-tests were one-sided given the directed hypotheses and that significant results in the opposite directions (target emotion ratings lower than non-target ratings) would not indicate that the expressions were perceived correctly. Paired Cohen’s *d* effect sizes were calculated via the ‘lsr’ package^30^ which calculates the paired effect size using the standard deviation of the differences akin to *dz*^33^. For post-hoc analyses, *t*-tests were conducted with an adjacent *p*-value of 0.002 (0.05/30 comparisons). For post-hoc tests, the rating of the target emotion was compared to the top-ranked non-target emotion, and a non-significant difference was used as an indicator that the target emotion was among the highest ranked emotions.

## Data Availability

Data, stimuli, and analysis are publicly available online at https://osf.io/b5rqz.
